# Annual Meeting of Governors and General Council

**Published:** 1917-05-05

**Authors:** 


### KING EDWARD'S HOSPITAL FUND FOR LONDON.

### Annual Meeting of Governors and General Council.

The annual meeting of the Governors and General Council of King Edward's Hospital Fund for London, to receive the accounts and the report of the General Council for the year 1916, was held at St. James's Palace on April 27, His Highness the Duke of Teck being in the chair.

Those present were: The Viscount Iveagh, the Speaker of the House of Commons (the Right Hon. James W. Lowther, M.P.), the Rev. W. L. Watkinson, D.D., the Chief Rabbi (the Rev. J. H. Hertz, D.D.), the Earl of Bessborough, the Viscount Knutsford, the Viscount Mersey, the Viscount Sandhurst, Lord Revelstoke, Lord Farquhar, Lord Burnham, Lord Stuart of Wortley, the Hon. Arthur Stanley, M.P., the Right Hon. Sir T. Vezey Strong, Sir William Church, Bt., Sir Walter Lawrence, Bt., Sir Francis H. Champneys, Bt., Sir Henry Burdett, Sir Owen Philipps, M.P., Sir William H. Bennett, Sir Everard Hambro, Sir William J. Collins, M.P., Sir Horace Marshall, Sir John Craggs, Sir John Tweedy, Mr. Frederick M. Fry (Hon. Secretary), Mr. John G. Griffiths (Hon. Secretary), Dr. Edwin Freshfield, Mr. W. J. H. Whittall.

### £143,000 Added to Reserve.

Lord Revelstoke (the Hon. Treasurer), in presenting the account of receipts and expenditure and the balance-sheet for the year ending December 31, 1916, said:

Your Highness, my Lords and Gentlemen,—I have the honour to submit the account of receipts and expenditure and the balance-sheet for the year 1916. The form of accounts has been slightly altered this year in order that items relating to the capital account may be more readily distinguished from those pertaining to the general fund, and the total disbursements from the balance carried to reserve. It is a matter for congratulation that, notwithstanding the exceptionally heavy calls entailed by the present state of affairs, the Fund has received such continued support as to enable it to put a substantial addition to reserve. This addition, which amounts to no less than £143,000, is due mainly to the receipt of £137,000 on account of the residue of the estate of the late Lady Wilton. Since the end of the year a further sum has been received, raising the total of the bequest to £163,000, thus more than justifying Sir Walter Trower's original estimate that the amount to be received by us in this connection would prove to be somewhat in excess of £150,000. Had it not been for this legacy, and for other large legacies and gifts received in the past, the Fund would not have had the power it possesses to-day of affording such invaluable assistance to the hospitals. It will not be forgotten that the confidence in the objects and methods of the Fund which inspired the donors and the testators of these large bequests and gifts was primarily due to the personal interest and to the supervision of His late Majesty King Edward and His present Majesty.

The net loss on the realisation of investments shown in the balance-sheet is due to a sale of the Fund's holding of American securities, which the Finance Committee considered it their duty to make in response to the invitation from H.M. Treasury, and the reinvestment of the proceeds in British Government securities. A large proportion of these American securities, which were of the very highest character, were originally presented to the Fund by generous donors. The result will be a small diminution in the income from investments, an income which, apart from this consideration, is still practically unaffected by the war.

The Speaker of the House of Commons moved the adoption of the accounts, which was seconded by the Viscount Sandhurst, and carried unanimously.

### The Year's Receipts.

Mr. John G. Griffiths (Hon. Secretary) presented the draft report of the Council for the year 1916, as follows:—

The total receipts for the year 1916 were £326,474 3s. 9d., of which £9,447 15s. 5d. were contributions to capital, and £317,026 8s. 4d. receipts on general account.

The general fund receipts were made up as follows: annual subscriptions, £25,403 3s. 6d.; donations, £5,837 1s. 5d.; contribution from League of Mercy, £15,000; from the estate of Isabella Countess of Wilton, £137,634 18s. 11d.; from the Lewis estate, £3,000; other legacies, £33,418 7s. 8d.; dividends and interest from investments, £96,532 16s. 10d.; from the Trustees of the Bawden Fund, £200; making a total of £317,026 8s. 4d.

#### Increased Grants for Maintenance.

The amount distributed was £170,000, which was £30,000 more than the amount applied to this purpose in 1915, and £12,500 more than the previous maximum appropriation before the war. In making these comparisons the supplementary distributions made in 1911 and 1915, resulting from the special donations from Sir Ernest Cassel, are omitted as being made after the original distributions were settled. Of the amount distributed, £162,500 was allocated to London hospitals and £7,500 to consumption sanatoria and convalescent homes taking London patients, as against £133,500 and £6,500 respectively in 1915. Of the £162,500 granted by the Fund to the London hospitals, £128,425 was given in aid of general maintenance, £15,000 to the reduction of debts on maintenance account, and £19,075 towards improvement schemes or in reduction of liabilities on such schemes undertaken before the war. The Fund has continued to encourage the hospitals in the policy of postponing all schemes of capital expenditure not exceptionally urgent or not already in hand at the outbreak of war, and the total grants in aid of new schemes were only £6,525. The increase in grants for maintenance over those made in 1915 amounted to £25,900.

#### Growing Needs Anticipated.

This large addition to the grants was, however, by no means in excess of the needs of the hospitals. The Fund made no serious increases in the distribution for maintenance in the first two years of the war, pending trustworthy evidence from the published accounts of the hospitals as to the extent of their greater burdens and the manner in which the different hospitals were being affected. This evidence began to appear in the accounts for 1915; and the policy of the Fund was justified by the fact that the Council was able, when the need was established, to come to the assistance of the hospitals concerned by substantial increases in the maintenance grants. The steady rise in prices of provisions and drugs, and in the cost of all departments, gives reason to fear that the needs of the hospitals have since continued and will continue to increase; and these matters will demand the consideration of the Fund in future distributions.

The £7,500 distributed by the Convalescent Homes Committee was appropriated, as to £5,535, to consumption sanatoria, and, as to £1,965, to convalescent homes. The grants to sanatoria enabled fifty-three beds to be reserved for the use of patients in London hospitals as compared with fifty-two last year.

The total sum distributed amongst hospitals, convalescent homes and consumption sanatoria during the last ten years (after deducting conditional grants to the extent of £6,500 that have been allowed to lapse), is £1,544,750. Since the foundation of the Fund a total amount of £2,268,416 has been distributed. The average annual distribution for the whole period of twenty years has been £113,420 16s. 5d.

Administrative Expenses Reduced.

During the year the amount spent on administration was £3,161 2s. 3d., or 19s. 4½d. per £100 of the total amount received, as compared with £3,205 16s. 1d., or £1 8s. 3d. per £100, last year. The corresponding percentage for the whole period since the inauguration of the Fund has been £1 4s. 3d., or less than 3d. in every £1 received, the average yearly outlay in this respect being £2,836 2s. 8d.

**Notable Subscriptions.**

His Majesty the King, patron of the Fund, has been graciously pleased to continue his annual subscription of £1,000. Her Majesty the Queen, Her Majesty Queen Alexandra, and His Royal Highness the Prince of Wales have also been pleased to subscribe to the Fund, as also have many other members of the Royal Family.

The subscribers will be glad to note that the list of contributions again includes annual subscriptions of £5,000 from Mr. Walter Morrison, £4,000 from the Drapers' Company, £1,000 from Lord Astor, and £1,000 from the Merchant Taylors' Company; besides an anonymous donation of £1,000. The Fund has also received annual subscriptions of £500 from Lord Iveagh, from Sir John R. Ellerman, Bt., from Mr. Robert Fleming, from Messrs. Morgan, Grenfell and Co., and from Messrs. N. M. Rothschild and Sons; and donations of the same amount from the Clothworkers' Company and from "S.D.R.S.D."

The League of Mercy this year has contributed £15,000, a larger sum than was received from the League in any of the last three years. The total amount contributed to the Fund by the League of Mercy since its foundation in 1899 now amounts to £245,000, apart from the sums awarded by the League to institutions outside the area of the Fund's distributions. The Council again express their appreciation of this very substantial benefit derived from the labours of a numerous band of voluntary workers.

Legacies received during the year include (in addition to the bequest of the late Isabella Countess of Wilton, and a further amount of £3,000 from the Lewis bequest) £20,193 19s. 5d. from the late Lady Brownlow, widow of Field-Marshal Sir Charles Brownlow; £10,000 given by the executors of the late Mr. Thomas Stephen Whitaker in the exercise of their discretionary powers; and £5,000 to capital from the late Rt. Hon. Sir Andrew Richard Scoble. The amount announced in the last annual report as received from the estate of the late Sir Julius Wernher has been increased by further receipts of £4,084 16s. 11d. during the year to £324,100 10s. 1d. This very generous legacy of one-twelfth of the residue of his estate, of which further amounts remain yet to be received, was left to be applied in augmentation of the capital account of the Fund; and the income the Fund is now receiving from this benefaction has contributed substantially to the distribution of the past year. The Fund has also received during the year the sum of £137,634 18s. 11d. on account of the residue of the estate of the late Lady Wilton. This legacy was estimated to realise £150,000, but since the end of the year further sums have been received raising the final total to £163,202 9s. 4d. The Fund desires to record its obligations to Sir Walter Trower in connection with the administration and realisation of the estate. The receipt of this important legacy, to the application of which no conditions were attached, largely influenced the Fund in the decision to make a material increase in the distribution.

Further Subscriptions and Donations Required and Deserved.

To maintain the annual distribution at £170,000 about £80,000 has to be raised each year from subscriptions, donations and legacies. The subscriptions and donations received in 1916 were £31,440 4s. 11d., an increase of £154 11s. 9d. over 1915. With the addition of the £15,000 from the League of Mercy, this source of revenue amounted to a little over £46,000 out of the £80,000 required. The uncertainty attaching to income from ordinary legacies is illustrated by the fact that, though in 1916 they amounted to £33,418 7s. 8d., in 1915 they had only produced £4,412 10s. 7d. The legacy from Lady Wilton thus provides a reserve against fluctuation in legacies during the coming years of possible stress.

Results of the War on Hospitals.

The Statistical Report on the expenditure of the London hospitals was again reduced in size this year in order to economise the consumption of paper and the employment of labour in printing. A fresh table of statistics showing the results of the war on hospitals and expenditure down to the end of 1915 was added to the prefatory note. This showed that the average number of beds in daily occupation by naval and military patients in 1915 was 2,378, of which about one-half were beds specially added for the purpose. The total additional ordinary expenditure in 1915, after deducting War Office payments, as compared with ordinary expenditure in 1913, was over £48,000, or nearly twice the amount by which the Fund's distribution to maintenance was increased. The causes of this increase in expenditure have operated with still greater force since the year 1915.

Each eligible hospital and consumption sanatorium which applied for a grant was inspected and reported upon, as hitherto, by two of the Fund's visitors, of whom one was a medical man and the other a layman.

The President of the Local Government Board, in pursuance of his powers under the Act, has again appointed Messrs. Deloitte, Plender, Griffiths and Co., chartered accountants, to audit the accounts of the Fund for the year. They have been the honorary auditors since January 1898, during which period they have rendered the Fund very valuable services.

The Viscount Iveagh formally moved the adoption of the report, which was seconded by Viscount Knutsford, who said they must all feel that it was very satisfactory that the Fund had been able to distribute such a very large sum, £170,000, amongst the hospitals. The report pointed out that "The total additional ordinary expenditure of hospitals in 1915, after deducting War Office payments, as compared with ordinary expenditure in 1913, was over £48,000." That was only for 1915; but the Fund would have to face the much larger expenditure in 1916 and the increasing cost in the present year.

The Viscount Mersey, in moving a vote of thanks to the Duke of Teck for presiding, said they all knew that His Highness had taken the greatest interest in this Fund for very many years past. The resolution was seconded by the Hon. Arthur Stanley, M.P., and carried unanimously.

The Duke of Teck having briefly acknowledged the vote of thanks, the proceedings terminated.

